# Short-term effects of highly-bioavailable curcumin for treating knee osteoarthritis: a randomized, double-blind, placebo-controlled prospective study

**DOI:** 10.1007/s00776-014-0633-0

**Published:** 2014-10-13

**Authors:** Yasuaki Nakagawa, Shogo Mukai, Shigeru Yamada, Masayuki Matsuoka, Eri Tarumi, Tadashi Hashimoto, Chieko Tamura, Atsushi Imaizumi, Jun Nishihira, Takashi Nakamura

**Affiliations:** 1 Department of Orthopedic Surgery, National Hospital Organization, Kyoto Medical Center, 1-1 Fukakusa Mukaihata-cho Fushimi-ku, Kyoto, 612-8555 Japan; 2 Theravalues Corporation, Tokyo, Japan; 3 Faculty of Medical Informatics, Hokkaido Information University, Ebetsu, Japan

## Abstract

**Background:**

We previously developed a surface-controlled water-dispersible form of curcumin and named it Theracurmin^®^ (Theracurmin; Theravalues, Tokyo, Japan). The area under the blood concentration–time curve of Theracurmin in humans was 27-fold higher than that of curcumin powder. We determined the clinical effects of orally administered Theracurmin in patients with knee osteoarthritis during 8 weeks of treatment.

**Methods:**

Fifty patients with knee osteoarthritis of Kellgren–Lawrence grade II or III and who were aged more than 40 years were enrolled in this randomized, double-blind, placebo-controlled, prospective clinical study. Placebo or Theracurmin containing 180 mg/day of curcumin was administered orally every day for 8 weeks. To monitor adverse events, blood biochemistry analyses were performed before and after 8 weeks of each intervention. The patients’ knee symptoms were evaluated at 0, 2, 4, 6, and 8 weeks by the Japanese Knee Osteoarthritis Measure, the knee pain visual analog scale (VAS), the knee scoring system of the Japanese Orthopedic Association, and the need for nonsteroidal anti-inflammatory drugs.

**Results:**

At 8 weeks after treatment initiation, knee pain VAS scores were significantly lower in the Theracurmin group than in the placebo group, except in the patients with initial VAS scores of 0.15 or less. Theracurmin lowered the celecoxib dependence significantly more than placebo. No major side effects were observed with Theracurmin treatment.

**Conclusion:**

Theracurmin shows modest potential for the treatment of human knee osteoarthritis.

## Introduction

Osteoarthritis, also referred to as degenerative joint disease, is a slow destructive process of the joints that affects millions of people worldwide. Although the exact biochemical cause of osteoarthritis remains unknown, the process usually begins when the joint structures are abnormal or the stress placed on joint surfaces is unusually high. Hip or knee osteoarthritis is a chronic condition that is usually treated with analgesics and nonsteroidal anti-inflammatory drugs (NSAIDs), but these drugs sometimes cause serious gastrointestinal and cardiovascular adverse events, especially with long-term use [1, 2]. Thus, there is a need for disease-modifying agents that not only decrease joint pain but also slow the progression of the condition.

Curcumin is a polyphenol extracted from turmeric, which has been safely used in foods such as curries for a long time [3]. Curcumin is a promising therapeutic food material because of its anti-inflammatory and antioxidative functions, and has long been used as an anti-inflammatory treatment in traditional Chinese and Ayurvedic medicine [3]. Curcumin regulates various biochemical and molecular pathways by modulating several molecular targets, including transcription factors, cytokines, enzymes, and genes regulating cell proliferation or apoptosis [4]. The anti-inflammatory effect of curcumin seems to be comparable with those of steroidal drugs and NSAIDs such as indomethacin and phenylbutazone [5]. Some studies have shown that curcumin’s anti-inflammatory properties are related to the suppression of prostaglandin synthesis through its effect on cyclooxygenase (COX) [6], a key enzyme responsible for the conversion of arachidonic acid to prostaglandins. Curcumin has also been shown to inhibit proteasome activity and induce apoptosis in human colon cancer cells in vitro and in vivo [7]. It has also been suggested that an important mechanism of curcumin is inhibition of NF-kB activation [8], which is a key event in the chronic inflammatory process. Given these findings, curcumin is expected to be effective for a range of diseases related to chronic inflammation, including cancer, cardiovascular disease, metabolic syndrome, Alzheimer disease, osteoarthritis, and other common diseases and aging conditions [3, 4, 9, 10]. Furthermore, it has been reported that curcumin can be a potent inhibitor of the production of inflammatory and catabolic mediators by chondrocytes [10]. Because osteoarthritis and related osteoarticular conditions of synovial joints are characterized by inflammation, curcumin’s biological actions in joint tissues may facilitate the development of clinically safe, orally administered therapeutic agents for treating joint diseases.

However, curcumin’s poor bioavailability has been an obstacle to realizing its beneficial health effects, because only a small amount of curcumin is absorbed with oral administration [11]. To overcome this bioavailability problem, we previously developed a surface-controlled water-dispersible curcumin and named it Theracurmin^®^ (Theracurmin: Theravalues, Tokyo, Japan) [12]. The absorption efficacy of Theracurmin was investigated and compared with that of curcumin powder. In rats, the area under the blood concentration–time curve (AUC) after oral administration of Theracurmin was found to be more than 40-fold higher than that of curcumin powder. With healthy human volunteers, the AUC of Theracurmin was 27-fold higher than that of curcumin powder. These findings demonstrated Theracurmin’s much higher bioavailability than those of currently available curcumin preparations. Thus, Theracurmin is believed to be useful for providing the clinical benefits of curcumin in humans.

The purpose of this study was to determine the clinical effects of orally administered Theracurmin in patients with knee osteoarthritis during 8 weeks of treatment. We hypothesized that Theracurmin ingestion for 8 weeks would improve the symptoms and functional abilities of patients with knee osteoarthritis.

## Materials and methods

A randomized, double-blind, placebo-controlled, prospective clinical study was conducted to test our hypothesis in two treatment groups: Theracurmin and placebo. A total of 50 patients with knee osteoarthritis confirmed by radiographic analysis were selected and enrolled in the study. Written informed consent was obtained from all subjects before participation. All procedures were reviewed and approved by the research ethics committee of our hospital, and this study was carried out in accordance with the World Medical Association’s Declaration of Helsinki.

The inclusion criteria were primary medial knee osteoarthritis patients over 40 years of age with Kellgren–Lawrence grades of II or III upon radiographic classification. The exclusion criteria were previous knee surgeries, knee injection treatment during the study, knee steroid injections within 2 months before the study, or other steroid administration within 4 weeks before the study. If patients needed NSAIDs during the study, oral celecoxib was prescribed, 2 pills per day (100 mg per pill). The other combined therapy we allowed was pain relief patches.

Theracurmin or placebo was administered orally twice a day for 8 weeks. Subjects in the Theracurmin group took 6 capsules of Theracurmin per day, which contained 180 mg of curcumin. Similarly, subjects in the placebo group took 6 placebo capsules per day, which had a similar shape and color to those of the Theracurmin capsules; the capsules were primarily made of starch, dextrin, and maltose. The subjects were requested to report the number of capsules remaining and celecoxib pills prescribed at their week 2, 4, 6, and 8 visits at our outpatient clinic for the compliance check.

Blood biochemistry analyses were performed before the study and after 8 weeks of each intervention. The patients’ knee symptoms were evaluated at 0, 2, 4, 6, and 8 weeks according to the following criteria: the Japanese Knee Osteoarthritis Measure (JKOM) [13], the knee pain visual analog scale (VAS) included in JKOM, and the knee scoring system of the Japanese Orthopedic Association (JOA) [14]. JKOM consists of 25 questions divided into 4 subcategories—pain and stiffness, condition in daily life, general activities, and health conditions—for patient self-assessment, and is based on the World Health Organization’s International Classification of Functioning, Disability, and Health. It is validated in the same manner as the Western Ontario and McMaster Universities’ Arthritis Index (WOMAC). The JOA scale evaluates four items: ability to walk (30 points), ability to climb up and down stairs (25 points), range of motion (35 points), and joint swelling (10 points). Each knee joint can achieve a maximum score of 100 points on the JOA scale. We used the improved JKOM, VAS, and JOA scores (the differences between the scores at each time point and those before the study). We evaluated adverse events and the number of celecoxib pills that the subjects needed during the 8-week period.

The two-sample one-sided *t* test was used to perform the statistical analysis of the VAS, JKOM, and JOA scores. The chi-square test was used to analyze the need for celecoxib. The level of statistical significance was set to a *P* value of <0.05.

## Results

**Fig. 1:**
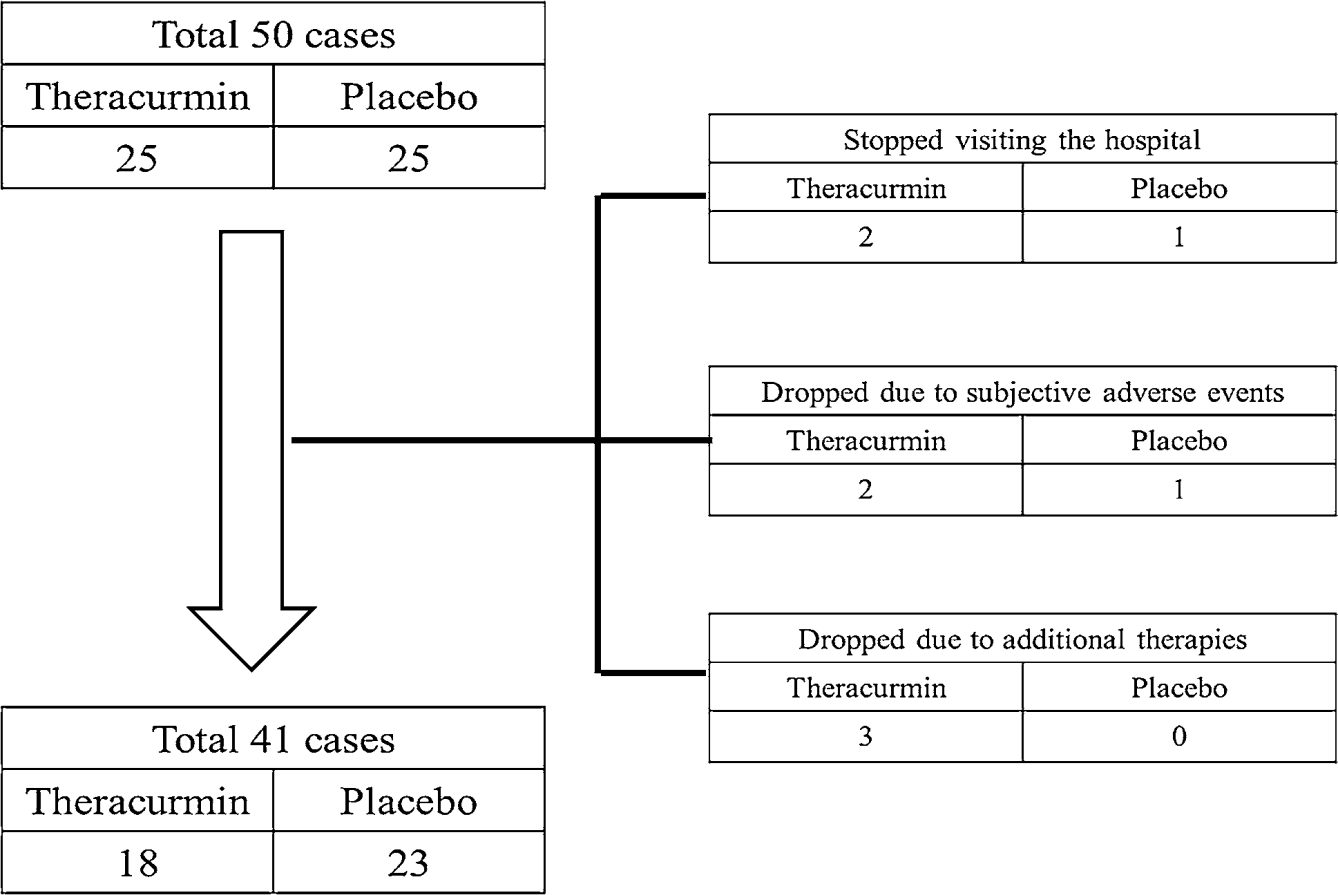
Study profile, including enrollments and dropouts. Seven patients in the Theracurmin group and two patients in the placebo group dropped out. Therefore, we analyzed 41 patients (18 patients in the Theracurmin group and 23 patients in the placebo group)

Drop-outs from the study are shown in Fig. 1. Three subjects (two and one in the Theracurmin and placebo groups, respectively) did not attend follow-up at weeks 1, 2, and 6. The other three patients discontinued participation in the study because of minor side effects. One patient in the Theracurmin group had a feeling of tachycardia and hypertension on day 50, and another patient in the Theracurmin group had redness of the tongue on day 6. In addition, one patient in the placebo group felt unwell on day 7. The other three cases in the Theracurmin group dropped out because they underwent intra-articular injection of hyaluronic acid on weeks 2, 2 (different patient), and 3. There were two Kellgren–Lawrence grade II cases and one Kellgren–Lawrence grade III case. The initial VAS scores in these three cases were 1.0, 0.54, and 0.45, and the improved VAS scores before intra-articular injection of hyaluronic acid were 0, 0.10, and 0.29, respectively. Therefore, we included 41 patients (18 and 23 patients in the Theracurmin and placebo groups, respectively) for further analysis.

The baseline characteristics of the study subjects in the two groups are shown in Table 1. Reflecting the general demographic profile of knee osteoarthritis, the majority of the subjects were female, with 77.8 % in the Theracurmin group and 78.3 % in the placebo group. The patient ages were also comparable between the two groups. The Kellgren–Lawrence criteria grade was used to quantify disease severity in order to effectively randomize disease status upon study entry between the groups. The majority of the subjects had grade II disease, with similar frequencies seen in the two groups. No statistical differences in the baseline characteristics were evident. Compliance was similar in the Theracurmin and placebo groups.

**Table 1:** Baseline characteristics of the study subjects in the two groups

	Theracurmin	Placebo	Total
Number of participants	18	23	41
Male (%)	22.2 (4/18)	21.7 (5/23)	22.0 (9/41)
Female (%)	77.8 (14/18)	78.3 (18/23)	78.9 (32/41)
Mean (SD) age (years)	71.9 (5.3)	66.1 (7.2)	68.7 (7.0)
Mean (SD) height (cm)	156.4 (7.0)	158.1 (7.6)	157.4 (7.3)
Mean (SD) body weight (kg)	61.5 (8.6)	62.1 (8.0)	61.8 (8.2)
Mean (SD) body mass index (kg/m^2^)	25.1 (2.7)	24.8 (2.3)	24.9 (2.4)
Osteoarthritis lesions			
Either left or right only (%)	72.2 (13/18)	56.5 (13/23)	63.4 (26/41)
Both left and right (%)	27.8 (5/18)	43.5 (10/23)	36.6 (15/41)
Kellgren–Lawrence classification for knee osteoarthritis			
II (%)	72.2 (13/18)	87.0 (20/23)	80.5 (33/41)
III (%)	27.8 (5/18)	13.0 (3/23)	19.5 (8/41)
Mean VAS score (SD)	0.52 (0.24)	0.42 (0.25)	0.47 (0.25)
Mean JOA score of each knee (SD)	82.5 (14.3)	84.8 (10.5)	83.8 (12.3)
Mean JKOM total score (SD)	35.5 (22.5)	27.0 (11.7)	307 (17.6)

No statistical differences in the baseline characteristics between the groups were evident

**Fig. 2:**
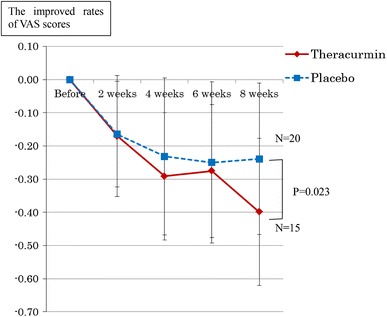
The improved VAS scores in the two groups are presented as means ± SDs. Except for the patients with initial VAS scores of 0.15 or less, the improved VAS scores were significantly larger in the Theracurmin group than in the placebo group at 8 weeks (*P* = 0.023)

Although the improved VAS scores were not significantly different between the two groups (*P* = 0.10) except in the patients with initial VAS scores of 0.15 or less, the improved VAS scores were significantly higher in the Theracurmin group than in the placebo group at 8 weeks (Fig. 2). The mean VAS scores at 0 and 8 weeks in all 41 cases were 0.52 and 0.20, respectively, in the Theracurmin group, and 0.42 and 0.21, respectively, in the placebo group. Except the patients with initial VAS scores of 0.15 or less, the mean VAS scores at 0 and 8 weeks were 0.60 and 0.20, respectively, in the Theracurmin group, and 0.47 and 0.23, respectively, in the placebo group. In each of the two groups there were three patients with initial VAS scores of 0.15 or less.

**Fig. 3:**
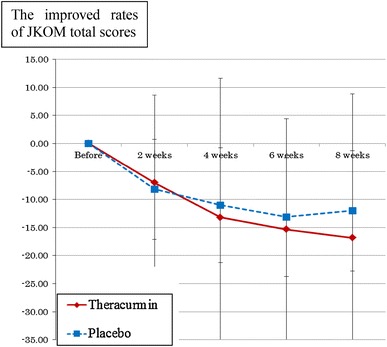
The improved JKOM total scores for the two groups are shown as the means ± SDs

There were no statistically significant differences in the JKOM scores between the two groups. However, the improved scores in the JKOM total score (Fig. 3) and in each JKOM subcategory tended to be higher in the Theracurmin group than in the placebo group, especially from 6 to 8 weeks in all 41 cases and 35 cases, except in the patients with initial VAS scores of 0.15 or less. There were no statistically significant differences in JOA total and subcategory scores (data not shown) between the two groups.

**Fig. 4:**
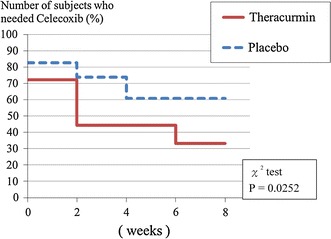
NSAID necessity in the two groups. At 8 weeks only, the ratio of patients who needed celecoxib was significantly smaller in the Theracurmin group than in the placebo group (*P* = 0.0252)

At 8 weeks only, NSAIDs were needed significantly less in the Theracurmin group than in the placebo group (Fig. 4).

At 8 weeks, all laboratory test results were similar to the baseline scores in both groups with the following exceptions: there were slight increases in triglyceride levels in some patients at week 8 in both groups (two patients in the Theracurmin group and four patients in the placebo group), slight increases in the level of creatinine (one case in the Theracurmin group), uric acid (one case in the placebo group), and amylase (one case in the placebo group) as well slight decreases in red blood cells (two cases in the Theracurmin group) and cholinesterase levels (one case in the Theracurmin group). These were all minor changes, and no serious adverse events were observed in either group during this study.

## Discussion

In this study, the knee pain VAS scores were significantly lower in the patients treated with Theracurmin than in those treated with placebo at 8 weeks among those who had initial VAS scores of >0.15. The same tendency was seen in the total JKOM scores and its subcategorical scores, including pain and stiffness in the knees, condition in daily life, general activities, and health conditions. Moreover, Theracurmin significantly lowered celecoxib dependence compared with placebo. These results suggest that Theracurmin may decrease the pain and discomfort of knee osteoarthritis and improve the patient’s general condition and quality of daily life. Because osteoarthritis is a slowly progressive and chronic condition that decreases quality of life, agents like Theracurmin, which is also a common food ingredient that is mildly effective with no major side effects, has modest potential for the treatment of human knee osteoarthritis.

The patients in the Theracurmin group took six capsules of Theracurmin per day, and each capsule contained 30 mg curcumin. We confirmed the safety of a Theracurmin dose of 150–210 mg that healthy humans could take in the phase 1 study. Therefore, six 30-mg capsules of Theracurmin per day were used in various clinical studies in our group, and no major side effects were observed.

In this study, the improved VAS scores between both groups were not significantly different, except in the patients with initial VAS scores of 0.15 or less. In each group, there were three patients with initial VAS scores of 0.15 or less. If the initial VAS score was small, the improved VAS score did not increase. This was because, when all 41 cases were considered, the improved VAS scores did not differ significantly between both groups. Three cases in the Theracurmin group dropped out because they underwent intra-articular injection of hyaluronic acid. The initial VAS scores in these three cases were 1.0, 0.54, and 0.45, and the improved VAS scores before intra-articular injection of hyaluronic acid were 0, 0.10, and 0.29. Our results indicate that Theracurmin was ineffective in some cases.

Some studies have suggested that curcumin has an effect on arthritis. Huang et al. [15] showed that curcumin dramatically attenuates the progression and severity of collagen-induced arthritis in mice and suppresses the production of the B-cell-activating factor belonging to the tumor necrosis factor family (a mechanism involved in rheumatoid arthritis). Banji D et al. [16] stated that combination treatment with methotrexate and curcumin had a significant anti-arthritic effect and protected against hematologic toxicity in rats. In addition to these anti-inflammatory effects, there curcumin seems to exert a chondroprotective action. For instance, Yang et al. [17] described novel pharmacological actions of curcumin on chondrocytes stimulated by advanced glycation end products. Kumar D et al. [18] also demonstrated that curcumin regulates the expression and secretion of various matrix metalloproteinases. Both the anti-inflammatory and chondroprotective effects of curcumin provide strong support for the potential effectiveness of curcumin treatment for osteoarthritis, and may explain the results of our present study. Further studies may be needed to show the chondroprotective effect of Theracurmin in knee osteoarthritis.

COX-2 inhibitors have been used as efficient anti-inflammatory agents or for osteoarthritis, although their cardiovascular toxicity can be problematic during long-time use. Lev-Ari et al. [19] reported a synergistic effect of celecoxib and curcumin that resulted in inhibition of the growth of osteoarthritic synovial adherent cells, which may be associated with increased induction of apoptosis, and stated that the synergistic effect was mediated by a mechanism that involved inhibition of COX-2 activity. In our study, Theracurmin decreased the need for the COX-2 inhibitor celecoxib. However, before they discontinued celecoxib, the subjects took both celecoxib and Theracurmin, so it is possible that there was a synergistic effect. Further study would be needed to clarify the best option among celecoxib alone, curcumin alone, and combined celecoxib and curcumin.

There have been three clinical reports that have examined the effects of curcumin in human osteoarthritic or rheumatic diseases. One pilot clinical study evaluated the safety and effectiveness of curcumin alone, diclofenac sodium alone, and their combination in patients with active rheumatoid arthritis [20]. Significantly greater improvement was shown by the curcumin group than by the diclofenac sodium group. More importantly, curcumin treatment was found to be safe and was not associated with adverse events. These results provided the first evidence for the safety and superiority of curcumin treatment in patients with active rheumatoid arthritis. With regard to curcumin’s effect on osteoarthritis, there have been two reported studies on the use of Meriva [21, 22], a complex of curcumin with soy phosphatidylcholine phytosomes, which have suggested that curcumin is efficacious for treating knee osteoarthritis. However, although these two studies were promising, both were open-label trials, not double-blind studies or randomized controlled trials with no restriction on NSAIDs, other painkillers, and other treatments prescribed by the general practitioners. The present study, which was a double-blind placebo-controlled trial, showed that even the placebo group showed statistically significant effects on all criteria, including those of the JOA scale, VAS, and JKOM (including all four JKOM subcategories), even though we only allowed celecoxib as a concomitant drug. Therefore, to evaluate the effects of specific compounds on knee osteoarthritis, we strongly believe that double-blind placebo-controlled studies should be conducted, which means that the effect seen in the Meriva studies may not be certain. In contrast, our study clearly showed Theracurmin’s effectiveness for treating knee osteoarthritis in a double-blind placebo-controlled study.

Several studies of Theracurmin have been conducted in animals and humans, and it has been suggested to be effective for treating cancer and cardiovascular conditions. For instance, two papers showing the clinical effectiveness of Theracurmin have been published. One paper suggested that regular endurance exercise combined with daily curcumin ingestion may decrease left ventricular afterload to a greater extent than monotherapy with either intervention alone in postmenopausal women [23]. The other paper showed that curcumin ingestion and aerobic exercise training increased flow-mediated dilation in postmenopausal women, which suggested that both can potentially improve age-related decline in endothelial function [24]. Similar to the present study, there were no major adverse effects of Theracurmin in the two previous studies, in spite of Theracurmin’s much higher bioavailability.

Patients with rheumatoid arthritis or osteoarthritis need novel anti-arthritic therapies because of possible gastrointestinal and cardiovascular adverse events caused by current therapies using NSAIDs and COX-2 inhibitors. There have been some in vitro and in vivo studies of arthritis treatment using natural health products or functional food materials. Ahmed et al. [25] suggested that epigallocatechin-3-gallate could decrease synovial hyperplasia, cartilage degradation, and bone resorption by modulating multiple targets in joints. Resveratrol may also prevent intervertebral disc degeneration, osteoarthritis-associated inflammation, chondrocyte apoptosis, and rheumatoid arthritis-related pannus formation, which are desirable goals in the treatment of osteoarthritis and rheumatoid arthritis [26]. In contrast to NSAIDs, curcumin has no gastrointestinal side effects and can even protect the gastric mucosa. Therefore, curcumin is thought to be beneficial in the management of chronic inflammation-related joint disease, including osteoarthritis [27]. However, because assessments of the outcomes of arthritis treatments are usually subjective, it is critical to confirm the effect of a test agent in double-blind placebo-controlled studies. For instance, glucosamine and chondroitin, which have often been used by arthritis patients, were found to be no more effective than placebo, and using then in combination did not decrease joint pain or have an impact on joint space narrowing [28]. In the present double-blind placebo-controlled study, we were able to provide the first evidence of the safety and superiority of curcumin treatment in patients with knee osteoarthritis. We also believe that we were able to obtain these results because of the high bioavailability of Theracurmin, which enhanced the health benefit of curcumin.

The limitations of our study were the small number of patients (*n* = 50) and short duration (8 weeks) of the treatment of knee osteoarthritis. This was a pilot study to determine the effect of Theracurmin on knee osteoarthritis, but we were fortunately able to observe significant outcomes with respect to improved VAS scores and decreased NSAID (celecoxib) necessity. Future studies may be needed to validate our findings in a large number of patients with knee osteoarthritis over a longer duration.

In our evaluations, we used JKOM, which was designed for Japanese patients with knee osteoarthritis, and did not use WOMAC, which is commonly used throughout the world; however, JKOM has been validated and is widely used in Japan.

In conclusion, we conducted a randomized, double-blind, placebo-controlled, prospective clinical study of the efficacy of Theracurmin, which is a highly bioavailable form of curcumin, in patients with osteoarthritis. Theracurmin was significantly effective in deceasing pain and NSAID necessity with no major adverse events. Theracurmin has modest potential for the treatment of human knee osteoarthritis in the future.
